# Review of "*Fish Defenses: Volume 2. Pathogens, Parasites and Predators*" by G. Zaccone, C. Perrière, A. Mathis, B. G. Kapoor (eds.)

**DOI:** 10.1186/1756-3305-3-24

**Published:** 2010-03-31

**Authors:** Pin Nie

**Affiliations:** 1 State Key Laboratory of Freshwater Ecology and Biotechnology, Institute of Hydrobiology, Chinese Academy of Sciences, Wuhan, Hubei Province 430072, PR China

## Abstract

Book review of "*Fish Defenses: Volume 2. Pathogens, Parasites and Predators*" by G. Zaccone, C. Perrière, A. Mathis, B. G. Kapoor (eds.)

## Review

Fish in nature live in a diversified aquatic world from fresh to brackish and from cold to tropical water, and they must have evolved adaptations to their biotic and abiotic environments. Fish are also important species for aquaculture, thus facing various kinds of diseases and/or stressors. This second volume of the series under the title, *Fish Defenses*, consists of eleven chapters, intended to "provide a general view of the current state of knowledge of fish defences with respect to pathogens, parasites and predators," as stated on the back-cover, and probably to their adaptations. This volume covers a variety of disciplines, including immunology, aquaculture and viral and bacterial fish diseases, parasitology, ecology including chemical ecology, evolution, behaviour, and predator-prey interactions.

The first two chapters devoted to mucosal immunity and antimicrobial peptides, written by well-known experts on the field, provide a good comparison on what is known in mammal and what is already known in fish, and provides some clues for further investigation. It is likely that these two chapters are too general and similar reviews may have been published elsewhere. The authors of the Chapter 2 have indeed written some excellent reviews on antimicrobial peptides in fish, as mentioned by themselves in this chapter. The title of this chapter, "Host Defense Peptides in Fish" may suit the needs of the volume well, but antimicrobial peptides may reflect the content presented in this chapter. In the first chapter, it is not known why the original reports on IgZ or IgT, which represent the most important progress in fish antibody study were not cited when dealing with these antibodies. Furthermore, a Canadian group of scientists identified morphologically dendritic cells in fish as early as in 2006, although further evidence was published very recently. So, the facts about fish dendritic cells can be updated.

In the following two chapters, fish immune responses to viral pathogens distributed mostly in Europe and North America, and some most recent progress in fish innate immunity, as well as streptococcal pathogens are well reviewed, with suggestions of reasonable strategies for the prevention of these diseases. This will be of considerable importance for further research and also for aquaculture practice.

The rest of the volume is ecological and/or evolutional. Chapters 5 and 8 are similar in title and indeed, some empirical examples were given in both, which might have been detected by editors. With limited literature regarding the behavioural defense and parental care, the mechanisms involved in such behaviour and function have not been clearly investigated so far. Instead, only a few hypotheses and suggestions can be proposed in these chapters. The unique predator-prey system, the opisthobranch slugs and predatory fish may be of interest to those working on predator-prey interactions. The speculation on pharmacology of substances in skin secretions is far from realistic and considerable research is needed before detergents or antimicrobial peptides can be used in our society. The work on chemical alarm cues was well summarized. However, with the exception of the immunological and disease parts, the volume could be better organized to avoid at least some overlapping in avoidance of parasites, and in some antimicrobial contexts.

The production quality of the book is in general very good. Some colour illustrations are quite clear and nice, such as those in Chapter 1. Spelling mistakes and/or citation problems are hardly found, with the only exception that (Bromage, 2005) which appeared on p25 was listed as 2006 in the reference list.

Despite the above criticisms, I consider that this volume contains a wealth of information and also references on fish defenses against viral, bacterial pathogens, and parasites as well as predators, and in each chapter background knowledge has been provided in good detail. This book will be undoubtedly consulted by both established and new scientists working in fish immunology, fish diseases, and in fish-parasite/pathogen and predator-prey relationships.

## Competing interests

The authors declare that they have no competing interests.

